# Dual-Genotype *Orientia tsutsugamushi* Infections, Hainan Island, China, 2023

**DOI:** 10.3201/eid3106.241967

**Published:** 2025-06

**Authors:** Yi Niu, Siqi Chen, Gaoyu Wang, Yijia Guo, Nan Ge, Xiaoyuan Hu, Chuanning Tang, Ruoyan Peng, Xiuji Cui, Biao Wu, Bo Wang, Yueping Wang, Dachuan Lin, Yongguo Du, Jasper Fuk-Woo Chan, Long Sun, Kwok-Yung Yuen, Liyuan Zhang, Feifei Yin

**Affiliations:** Hainan Medical University, Haikou, China (Y. Niu, S. Chen, G. Wang, Y. Guo, N. Ge, X. Hu, C. Tang, R. Peng, X. Cui, B. Wu, B. Wang, Y. Wang, D. Lin, Y. Du, L. Sun, L. Zhang, F. Yin); University of Hong Kong, Hong Kong, China (J. F.-W. Chan, K.-Y. Yuen)

**Keywords:** *Orientia tsutsugamushi*, scrub typhus, bacteria, tropical diseases, China

## Abstract

We report 3 cases of dual-genotype *Orientia tsutsugamushi* infection in Hainan Island, China. Patients exhibited diverse clinical manifestations, including afebrile illness and multiorgan involvement, highlighting the complexity associated with genetic diversity in scrub typhus. Clinicians should maintain heightened suspicion for atypical scrub typhus manifestations in endemic regions.

Scrub typhus, caused by the obligate intracellular bacterium *Orientia tsutsugamushi*, is a significant but underrecognized tropical disease endemic found mainly throughout the Asia-Pacific region (*1*). Scrub typhus places approximately 1 billion persons at risk and causes illness in 1 million persons each year (*2*). Extensive genetic diversity is a hallmark of *O. tsutsugamushi*, influencing disease severity and complicating vaccine development and diagnostic accuracy (*3*). We describe 3 cases from Hainan Island, China, that illustrate the clinical variability and diagnostic challenges associated with dual-genotype infections and underscore the need for further investigation into their clinical implications.

## The Study

Patient 1 was a 55-year-old male farmer from Lingao County in Hainan Province, China (Table), who sought treatment for symptoms that included a 5-day history of fever peaking at 39.8°C, along with chills, headache, and fatigue. On September 12, 2023, he was admitted to the Infectious Diseases Department, Second Affiliated Hospital of Hainan Medical University (Haikou, China). At admission, laboratory test results showed markedly elevated inflammatory markers and liver enzymes: high-sensitivity C-reactive protein 122.70 mg/L (reference range <10 mg/L), neutrophil percentage 84.1% (reference range 40%–70%), alanine aminotransferase 301 U/L (reference range <40 U/L), aspartate transaminase 384 U/L (reference range <40 U/L), and direct bilirubin 7.0 μmol/L (reference range <5 μmol/L). In addition, interleukin 6 was markedly elevated at 156.00 pg/mL (reference range <7 pg/mL) and D-dimer was 100.39 μg/mL (reference range <0.5 μg/mL). 

**Table:** Clinical and laboratory features of patients in study of dual-genotype *Orientia tsutsugamushi* infections, Hainan, China, 2023*

Patient	Age, y/sex	Clinical status at admission	Site of eschar	Disease severity	Genotypes (ratio)	Hospital stay, d
1	55/M	5-day history of fever, headache, fatigue	NA	Moderate	Karp_B + JG_C (1:2)	6
2	49/M	10-day history of fever, cough, dyspnea	Below clavicle	Severe (ICU)	Karp_A + JG_C (17:2)	16
3	64/M	5-day history of ataxia, weakness (afebrile)	Hip	Severe (shock)	Karp_B + Kato_B (1:1)	6

*ICU, intensive care unit; NA, not applicable.

We determined the illness to be scrub typhus based on clinical manifestations and results from real-time PCR testing targeting the *O. tsutsugamushi* 47-kDa and 56-kDa type-specific antigen genes (Zybio Inc., https://www.zybio.com) and serologic testing for IgM and IgG using a Gold-immuno-chromatographic assay kit (Beijing Wantai, https://www.ystwt.cn). We promptly administered intravenous doxycycline (0.2 g in 250 mL normal saline daily) plus supportive care, and the patient’s condition gradually improved. He was discharged 6 days after admission.

Patient 2 was a 49-year-old male farmer from Wanning City, Hainan Province, whose symptoms included a 10-day history of high-grade fever (up to 40°C), cough with yellow sputum, and chest tightness (Table). His symptoms worsened 2 days before admission, with recurrence of those symptoms plus shortness of breath and urinary frequency. Admitted to the emergency intensive care unit (ICU), First Affiliated Hospital of Hainan Medical University (Haikou, China), on June 14, 2023, the patient received an initial diagnosis of severe pneumonia. Laboratory tests revealed severe inflammation and multiorgan involvement: high-sensitivity C-reactive protein 183.74 mg/L, neutrophil percentage 75.6%, leukocyte count 17.94 × 10^9^ cells/L (reference range 4–10 × 10^9^ cells/L), alanine aminotransferase 77 U/L, aspartate transaminase 150 U/L, D-dimer 11.81 μg/mL, albumin 19.6 g/L (reference range 35–50 g/L), arterial oxygen partial pressure/fractional inspired oxygen ratio 284 mm Hg (reference range >300 mm Hg), and platelet count 67 × 10^9^/L (reference range 150–400 × 10^9^/L). Imaging revealed bilateral pulmonary infiltrates, mild pleural effusion, enlarged axillary lymph nodes, and splenomegaly. Investigation revealed a 0.3 cm × 0.5 cm eschar below the right clavicle, strongly suggesting scrub typhus as the underlying cause. We later confirmed *O. tsutsugamushi* infection by real-time PCR and serologic testing. Oral doxycycline (0.2 g/d) and supportive treatment led to improvement by the third day of treatment. The patient was discharged 16 days after admission.

Patient 3 was a 64-year-old male farmer from Chengmai County, Hainan Province, who sought treatment for a 5-day history of unsteady gait, limb weakness, and fatigue (Table). He had a 40-year history of epilepsy, managed with valproic acid and oxcarbazepine. The patient was admitted to the Neurology Department, Second Affiliated Hospital of Hainan Medical University, on November 23, 2023. Examination showed impaired articulation and a positive Romberg sign, but no fever (36.8°C). Laboratory tests indicated elevated inflammatory markers and mild hepatic injury: high-sensitivity C-reactive protein 71.06 mg/L, alanine aminotransferase 54 U/L, aspartate transaminase 86 U/L, interleukin 6 127.00 pg/mL, D-dimer 5.47 μg/mL, and platelet count 62 × 10^9^/L. Computed tomography of the patient’s chest revealed mild, ground glass opacities in multiple lung segments, bilateral pleural thickening, mild pleural effusion, and enlarged axillary lymph nodes. Given the initial signs and symptoms of thrombocytopenia, liver dysfunction, and pneumonitis, we treated the patient with antiepileptic drugs and ceftriaxone, which imparted minimal effect. On the third hospital day, attending staff noted a 0.3 cm × 0.4 cm eschar on the patient’s hip. Subsequent real-time PCR analysis and IgM/IgG serologic testing for *O. tsutsugamushi* yielded positive results, thereby confirming a diagnosis of scrub typhus. We then treated the patient with intravenous doxycycline (0.2 g in 250 mL normal saline daily), along with supportive treatments that included inotropic support and antiepileptic therapy. His condition improved within 3 days, and he was discharged 6 days after admission.

All 3 patients with scrub typhus tested positive by real-time PCR, IgM and IgG serology, and nested PCR targeting the 483-bp fragment of the 56-kDa type-specific antigen gene (*4*). Sanger sequencing showed overlapping nucleotide peaks, indicating mixed infection. Cloning and subsequent sequencing of individual amplicons confirmed dual-genotype *O. tsutsugamushi* infections. Patient 1 was co-infected with Karp B (Karp group) and JG_C (Gilliam group) genotypes at a 1:2 ratio (Karp B: 5/15 clones; JG_C: 10/15 clones), with 74.43% nucleotide sequence identity between them (Figure). Patient 2 carried Karp A (Karp group) and JG_C genotypes in a 17:2 ratio (Karp A: 17/19 clones, JG_C: 2/19 clones) at 74.25% identity. Patient 3 was co-infected with Karp B and Kato_B (Kato group) genotypes in a 1:1 ratio (Karp B: 9/18 clones, Kato_B: 9/18 clones) at 72.87% identity.

**Figure:**
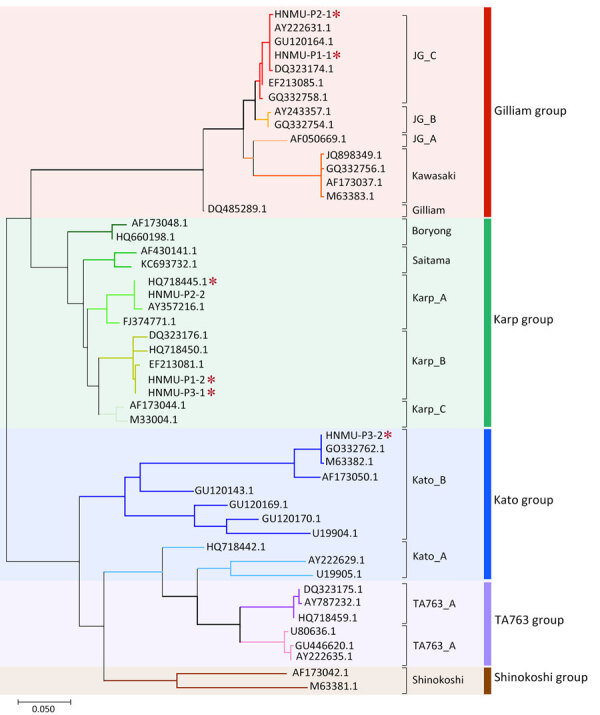
Phylogenetic analysis of *Orientia tsutsugamushi* based on partial 56-kDa type-specific antigen gene sequences from a study of dual-genotype *O. tsutsugamushi* infections, Hainan Island, China, 2023. Phylogenetic tree was constructed using partial gene sequences from whole blood samples of 3 patients with scrub typhus (red asterisks) and reference sequences representing dominant genotypes retrieved from GenBank (accession numbers indicated). We performed the analysis with MEGA X software (https://www.megasoftware.net) using the maximum-likelihood method and 1,000 bootstrap replicates. Color shading and vertical bars at right indicate genotypes.

All 3 patients were middle-aged, male farmers (mean age 56 ± 7.55 years) with recent outdoor exposure, consistent with typical risk factors for scrub typhus. We discovered an eschar on 2 of the patients, a characteristic clinical feature of the disease. The severity of illness varied widely among the 3 case patients: 1 patient developed severe scrub typhus requiring ICU care; another had a relatively mild, afebrile course. Despite differences in severity, all 3 case patients shared common features, including pulmonary involvement and liver dysfunction, markedly elevated inflammatory markers, and thrombocytopenia. Among the 3 patients, patient 2 exhibited the highest proportion of the Karp_A genotype and required ICU admission, indicating greater disease severity (Table). Previous studies have associated the Karp genotype with more severe clinical manifestations and higher bacterial loads (*5*–*7*). Although limited by sample size, our preliminary observations suggest that genotype dominance in dual-genotype infections may influence disease severity, warranting larger-scale studies.

## Conclusions

Dual-genotype *O. tsutsugamushi* infections represent a complex and underexplored aspect of scrub typhus epidemiology (*8*–*13*). Our study highlights dual-genotype *O. tsutsugamushi* infections in 3 patients from Hainan Island, China, and emphasizes the genetic complexity and clinical variability associated with scrub typhus. We observed Karp-group strains consistently present alongside Gilliam and Kato strains, reflecting dominant regional circulation patterns (*5*). Despite varied clinical severities, all patients exhibited common features, namely pulmonary involvement, hepatic dysfunction, elevated inflammatory markers, and thrombocytopenia. The presence of multiple genotypes within individual infections, confirmed through sequencing and cloning, underscores the diagnostic and therapeutic challenges associated with scrub typhus. Our findings necessitate further studies to elucidate the influence of dual-genotype infections on disease progression, clinical outcomes, and treatment efficacy. Clinicians should maintain heightened suspicion for atypical scrub typhus manifestations in endemic regions.
